# The association of eicosanoids with lung structure and function: Findings from the Multi-Ethnic Study of Atherosclerosis lung study and Framingham Heart Study

**DOI:** 10.1371/journal.pone.0351692

**Published:** 2026-06-30

**Authors:** Mythri Ambatipudi, Jenna N. McNeill, Athar Roshandelpoor, Mona Alotaibi, Louisa A. Mounsey, Eric Hoffman, George O’Connor, Seung Hoan Choi, Norrina B. Allen, R. Graham Barr, Mohit Jain, Susan Cheng, Jennifer E. Ho

**Affiliations:** 1 CardioVascular Institute and Division of Cardiology, Department of Medicine, Beth Israel Deaconess Medical Center, Boston, Massachusetts, United States of America; 2 Division of Pulmonary, Allergy, and Critical Care Medicine, Department of Medicine, Duke Hospital, Durham, North Carolina, United States of America; 3 Division of Pulmonary and Critical Care and Sleep Medicine, Department of Medicine, University of California San Diego, La Jolla, California, United States of America; 4 Department of Radiology, University of Iowa Carver College of Medicine, Iowa City, Iowa, United States of America; 5 Pulmonary Center, Boston University School of Medicine, Boston, Massachusetts, United States of America; 6 Department of Biostatistics, Boston University School of Public Health, Boston, Massachusetts, United States of America; 7 Cardiovascular Disease Initiative, Broad Institute of Harvard and the Massachusetts Institute of Technology, Cambridge, Massachusetts, United States of America; 8 Department of Preventive Medicine, Northwestern University Feinberg School of Medicine, Chicago, Illinois, United States of America; 9 Department of Medicine, Columbia University Irving Medical Center, New York, New York, United States of America; 10 Department of Epidemiology, Mailman School of Public Health, Columbia University, New York, New York, United States of America; 11 Department of Medicine and Department of Pharmacology, University of California San Diego, La Jolla, California, United States of America; 12 Department of Cardiology, Smidt Heart Institute, Cedars-Sinai Medical Center, Los Angeles, California, United States of America; Southern Illinois University School of Medicine, UNITED STATES OF AMERICA

## Abstract

**Background:**

Eicosanoids are bioactive signaling lipids that have roles in airway remodeling, smooth muscle hypertrophy, emphysema and pulmonary fibrosis via mediation of pro- and anti-inflammatory pathways. Specific eicosanoids have been associated with lung diseases such as asthma and pulmonary fibrosis, yet their association with lung function more broadly is not completely understood. We aimed to investigate the association of eicosanoids and related metabolites with early changes in lung function and structure.

**Methods:**

We performed comprehensive profiling of over 250 eicosanoids and eicosanoid-related metabolites using directed non-targeted mass spectrometry in the Multi-Ethnic Study of Atherosclerosis (MESA) Lung Study with independent validation in the Framingham Heart Study (FHS). We performed cross-sectional analysis of the associations between metabolites and lung function as assessed by spirometry and quantitative lung measures on computed tomography (CT).

**Results:**

Among 3384 participants (mean age 63 ± 10 years, 51% women), 51 metabolites were associated with lung function in MESA Lung (22 with % predicted FEV_1_, 18 with % predicted FVC, and 25 with FEV_1_/FVC ratio), with 24 validated among FHS participants. Of these 51 metabolites, 27 were associated with obstructive lung physiology, including linoleic acid derivatives (9-HODE) and other long-chain fatty acids (hydroxyhexadecanoic acid, hydroxyoctadecanoic acid) associated with higher odds. Fourteen metabolites were associated with restrictive physiology, including putative dihydroxy-20:3 and an LTB3 analog associated with lower odds, and omega-3 fatty acids (EPA, stearidonic acid) associated with higher odds.

**Conclusions:**

Specific eicosanoids and eicosanoid-related metabolites including linoleic acid derivatives and long-chain fatty acids were associated with obstructive, and leukotrienes and omega-3 fatty acids with restrictive lung physiology. These findings highlight bioactive lipids involved in both pro- and anti-inflammatory pathways as potential influencers of lung function and may serve as future therapeutic targets early in lung disease development.

## Introduction

Chronic lung disease including chronic obstructive pulmonary disease (COPD), emphysema, pulmonary fibrosis, and asthma have serious impacts on health, quality of life, and mortality [1–5]. In 2017, chronic respiratory diseases affected 545 million people, accounting for 3.9 million deaths worldwide [6]. In light of the continued obesity epidemic, worsening pollution, and an aging population, the global burden of chronic lung disease is projected to increase [7, 8].

While there are many patient and population factors contributing to development of lung disease, inflammation is a key common pathway underlying both obstructive and restrictive diseases [9]. Eicosanoids are a class of bioactive lipids derived from arachidonic acid (AA) that govern upstream initiation of pro- and anti-inflammatory activity [10]. Prior studies have examined specific eicosanoid metabolites including leukotrienes and prostaglandins as important mediators of lung disease including COPD, emphysema, asthma, pulmonary fibrosis, and pulmonary hypertension where they are thought to incite inflammation, regulate epithelial cell function, and promote fibrosis [11–14]. In addition, leukotrienes contribute to airway remodeling via increased bronchial smooth muscle and fibroblast proliferation [11]. Indeed, targeting key eicosanoid pathways has proven effective in lung disease, and established therapies include singulair (leukotrienes antagonist) and zileuton (5-lipoxygenase inhibitor) for asthma, and synthetic prostacyclins (iloprost) and others for pulmonary arterial hypertension [15–18]

While specific eicosanoids have been implicated in lung disease, prior studies are limited to analysis of only dozens of metabolites, providing incomplete understanding of a broader bioactive lipid profile. We recently demonstrated the ability to reproducibly ascertain over 250 eicosanoid and related metabolites using a novel directed nontargeted liquid chromatography mass spectrometry (LC-MS)-based approach [19]. In this study, we leveraged this platform to investigate the association of eicosanoids and eicosanoid-related metabolites with early changes in lung function and structure across two major community-based cohorts. By examining population-based samples, we sought to examine the role of eicosanoids and eicosanoid-related metabolites as upstream regulators of inflammation to determine their role in early disease and inform potential preventive therapeutic targets.

## Methods

### Study sample

The Multi-Ethnic Study of Atherosclerosis (MESA) recruited 6814 adults 45–84 years old who self-reported white, Black, Hispanic or Asian race/ethnicity and were free of clinical cardiovascular disease (CVD) from 2000–2002 [20]. We included participants from examination cycle 2 (2002–2004) who provided plasma samples for eicosanoid analysis (n = 5457). We excluded those with prevalent CVD (heart failure, myocardial infarction) (n = 39), end-stage kidney disease (ESKD) (n = 19), or missing key clinical covariates (n = 253), yielding 5150 participants. The MESA Lung Study performed spirometry in examination cycles 3–4 (2004–2007) among participants with genetic consent, endothelial function measures and with oversampling of Asian, 3489 of whom had eicosanoid measures [21]. 104 had incomplete or low-quality spirometry data, leaving 3385 participants. One participant was subsequently identified as a blood sample outlier, leaving 3384 participants (S1 Fig).

We performed external validation among the Framingham Heart Study (FHS) Offpsring examination cycle 8 (2005–2008) participants. Of the 2394 participants with assayed plasma samples, we excluded those with prevalent CVD (n = 32), ESKD (n = 29), or missing clinical covariate data (n = 11). Of the remaining 2321 patients, 2071 had complete spirometry measurements between 2005–2008.

Our study involved secondary use study of existing deidentified and coded data accessed on 08/01/2022 and was approved by the Beth Israel Deaconess Medical Center institutional review board. Investigators did not have access to information that could identify individual participants during or after data collection. The original ‘parent studies’ included MESA and FHS, and all participants gave written informed consent with respective MESA and FHS institutional review board approvals (S1 Checklist).

### Clinical assessment

Medical history, physical exam, and laboratory data were available for both MESA and FHS participants. Body mass index (BMI) was defined as weight/height^2^ (kg/m^2^) and ESKD was defined as estimated glomerular filtration rate (eGFR) < 30 ml/min/1.73 m^2^. Smoking status was self-reported.

### Pulmonary function testing

The MESA Lung study performed pre-bronchodilator spirometry using rolling barrel spirometers (OMI systems) following previously described ATS/ERS guidelines [22]. Spirometry and diffusing capacity of the lungs for carbon monoxide (DLCO) measurements were collected for FHS participants using the Collins Comprehensive Pulmonary Laboratory system (nSpire Health Inc., Longmont, CO, USA) [23]. Percent predicted FEV_1_ and FVC (PPFEV_1_, PPFVC) were calculated using published reference values and equations [24, 25]. As per prior manuscripts, we utilized predicted spirometry values based on Hankinson equations. Restrictive physiology was defined as FEV_1_/FVC > 0.7 and PPFVC<80% [26, 27]. Participants with FEV_1_/FVC < 0.7 and PPFEV_1_ between 80% to 100% were classified as Global Initiative for Obstructive Lung Disease (GOLD) grade 1 obstructive physiology while those with FEV_1_/FVC < 0.7 and PPFEV_1_ < 80% were classified as GOLD grade 2–4 obstructive physiology (combined into one due to small sample sizes in grades 3–4) [27–29].

### Lung imaging

MESA participants underwent low-dose computed tomography (CT) imaging for coronary artery calcium at exam 1 (2000–2002) [30]. Images were analyzed for high attenuation areas (HAA), defined as percentage of imaged lung volume with CT attenuation between −600 and −250 Hounsfield Units (HU) [31, 32], and percent emphysema defined as percentage of imaged lung voxels below 950 HU. [32–34] At exam 5 (2010–2012), participants underwent full-lung CT scans [35] allowing for identification of interstitial lung abnormalities (ILA), defined as presence of ground-glass, reticular abnormality, diffuse centrilobular nodularity, honeycombing, traction bronchiectasis, non-emphysematous cysts or architectural distortion in ≥5% of nondependent lung regions [36, 37]. Continuous HAA was created through log2-transformation of percent HAA [32]. ILA was analyzed as a binary variable: absence of ILA versus indeterminate or definitive ILA [37].

### Plasma metabolite profiling

Eicosanoid profiling for MESA and FHS was performed at the University of California, San Diego. Plasma samples were drawn after ≥8 hours of fasting and stored at −80°C. Samples underwent at most one freeze-thaw cycle prior to analysis. As previously described, eicosanoids and related metabolites were extracted and assayed via a directed, non-targeted LC-MS method developed and validated by Watrous et al. in a subset of 1500 participants drawn from the 2394 FHS participants included in this present study [19, 38]. Commercially-available standards and MS/MS fragmentation patterns were used to annotate metabolites, which were validated using spectral fragmentation pattern networking and manual annotation.

Due to informative missingness indicating concentrations below detectable threshold, missing values were imputed as 25% of the minimum value of that metabolite across participants. Low-abundance metabolites with >90% of data missing across participants were excluded. A total of 784 eicosanoids and related metabolites (98% of total) were included in subsequent MESA analyses. Metabolite aligment between MESA and FHS was determined via mass spectrometry peak matching using m/z (mass-to-charge ratios) and retention times. A total of 454 metabolites were present in both MESA and FHS and considered for external validation.

### Statistical analysis

Baseline characteristics were summarized by cohort, with continuous variables reported as mean (SD) or median (IQR) and categorical variables as number (percentage). Metabolite concentrations were natural log-transformed due to right skewness and standardized to a distribution with a mean of 0 and standard deviation of 1. In principal component analysis, an extreme outlier was identified and excluded. In primary analyses, we investigated associations between metabolites and lung function as measured by PPFEV_1_, PPFVC, and FEV_1_/FVC using multivariable linear regression models. Models were adjusted for age, sex, plate number, race, BMI, diabetes, hypertension, aspirin use, and statin use. Secondary models additionally adjusted for smoking status. False discovery rate (FDR)-adjusted q < 0.05 was deemed significant for both MESA and the FHS validation sample. Significant metabolites were also tested for significant associations with DLCO in FHS (not available in MESA).

In secondary analyses, we examined the association of metabolites with obstructive and restrictive physiology, with the comparator group defined as individuals who did not meet obstructive or restrictive criteria and FDR-adjusted q < 0.1 deemed signficant. Logistic regression models were adjusted for the same covariates as in primary analyses. We used FDR-adjusted q < 0.1 to validate results in FHS. Among the 2233 MESA participants with lung imaging available, we examined the association of spirometry-associated metabolites with percent HAA, presence of ILA, and percent emphysema. We used multivariable regression models adjusted for covariates listed above, in addition to CT scanner type and wt220 (whether weight>220 lbs in Exams 1–4, MDCT scanners only). P < 0.05 was deemed signficant.

In exploratory analyses, we investigated the role of eicosanoids and related metabolites as mediators of the interaction between smoking status and obstructive physiology. Mediation analysis was performed using the “mediation” package in R, with eicosanoids as mediators, obstructive physiology as the outcome, and smoking status (never smoker versus past/current smoker) as the exposure. Magnitudes and directionalities of associations between exposure and mediator, mediator and outcome, and exposure and outcome were examined, as well as sizes of mediation effects. Significant mediation was defined as mediation p < 0.05.

All analyses were performed in R studio using R version 4.2.1.

## Results

Among 3384 MESA participants (mean age 63 ± 10 years, 51% female), 44% had hypertension, 13% diabetes mellitus, and 11% were current smokers, with mean BMI 28.1 ± 5.3 kg/m^2^. The majority had normal lung physiology with median PPFEV_1_ of 100% (Q1 92%, Q3 110%), PPFVC of 98% (Q1 90%, Q3 108%), and FEV_1_/FVC of 0.78 (Q1 0.74, Q3 0.81) (Table 1). A total of n = 989 (29.2%) had abnormal pulmonary physiology, with 18.6% obstructive (8.7% grade 1, 9.9% grade 2–4 obstruction) and 10.7% restrictive physiology.

**Table 1:** Clinical characteristics of MESA and FHS participants.

	MESA				FHS			
	Normal N = 2395	Restriction N = 361	Grade 1 Obstruction (N = 294)	Grade 2–4 Obstruction (N = 334)	Normal N = 1467	Restriction N = 94	Grade 1 Obstruction N = 273	Grade 2–4 Obstruction N = 237
Clinical Measures								
Age, years	62 (10)	63 (9)	67 (9)	66 (9)	66 (9)	65 (9)	67 (9)	69 (8)
Female, N (%)	1272 (53)	206 (57)	99 (34)	138 (41)	853 (58)	50 (53)	132 (48)	141 (60)
Current smoker, N (%)	190 (8)	44 (12)	49 (17)	71 (21)	75 (5)	16 (17)	33 (12)	47 (20)
Former smoker, N (%)	875 (37)	112 (31)	133 (45)	168 (50)	455 (31)	23 (25)	116 (43)	117 (49)
Pack years smoking, years	9 (17)	14 (29)	21 (28)	27 (31)	11 (17)	16 (22)	22 (24)	33 (27)
BMI, kg/m^2^	28 (5)	30 (6)	27 (4)	28 (5)	28 (5)	32 (7)	28 (5)	28 (6)
Hypertension, N (%)	978 (41)	187 (52)	139 (47)	175 (52)	977 (67)	79 (84)	195 (71)	175 (74)
Diabetes mellitus, N (%)	288 (12)	81 (22)	25 (9)	50 (15)	166 (11)	27 (29)	32 (12)	40 (17)
Spirometry Measures, median (Q1, Q3)								
FEV1, % predicted	100 (92, 110)	76 (68, 81)	89 (84, 94)	69 (58, 76)	103 (95, 112)	75 (70, 80)	90 (85, 94)	69 (62, 76)
FVC, % predicted,	98 (90, 108)	73 (68, 77)	102 (96, 108)	84 (75, 91)	104 (95, 114)	74 (70, 77)	103 (99, 108)	86 (79, 94)
FEV1/FVC ratio	0.78 (0.74, 0.81)	0.79 (0.76, 0.83)	0.67 (0.63, 0.69)	0.63 (0.55, 0.67)	0.75 (0.72, 0.78)	0.77 (0.73, 0.80)	0.66 (0.63, 0.68)	0.61 (0.55, 0.65)
DLCO^1^					22 (18, 27)	19 (15, 22)	21 (18, 26)	18 (15, 22)
CT measures (n = 2233)^2^								
% Emphysema	4 (4)	2 (2)	6 (4)	6 (5)				
% High attenuation areas	7 (4)	9 (7)	6 (3)	6 (2)				
Interstitial lung abnormalities, N (%)	401 (25)	52 (24)	69 (39)	64 (31)				

Values are mean (SD) unless specified.

^1^1972 FHS participants had DLCO measures (Normal: 1441; Restrictive: 87; Obstructive: 259 grade 1, 273 grade 2–4)

^2^2233 MESA participants had CT measures (Normal: 1630; Restrictive: 216 restrictive; Obstructive: 178 grade 1, 209 grade 2–4)

### Associations of eicosanoids with spirometry variables

**Fig 1:**
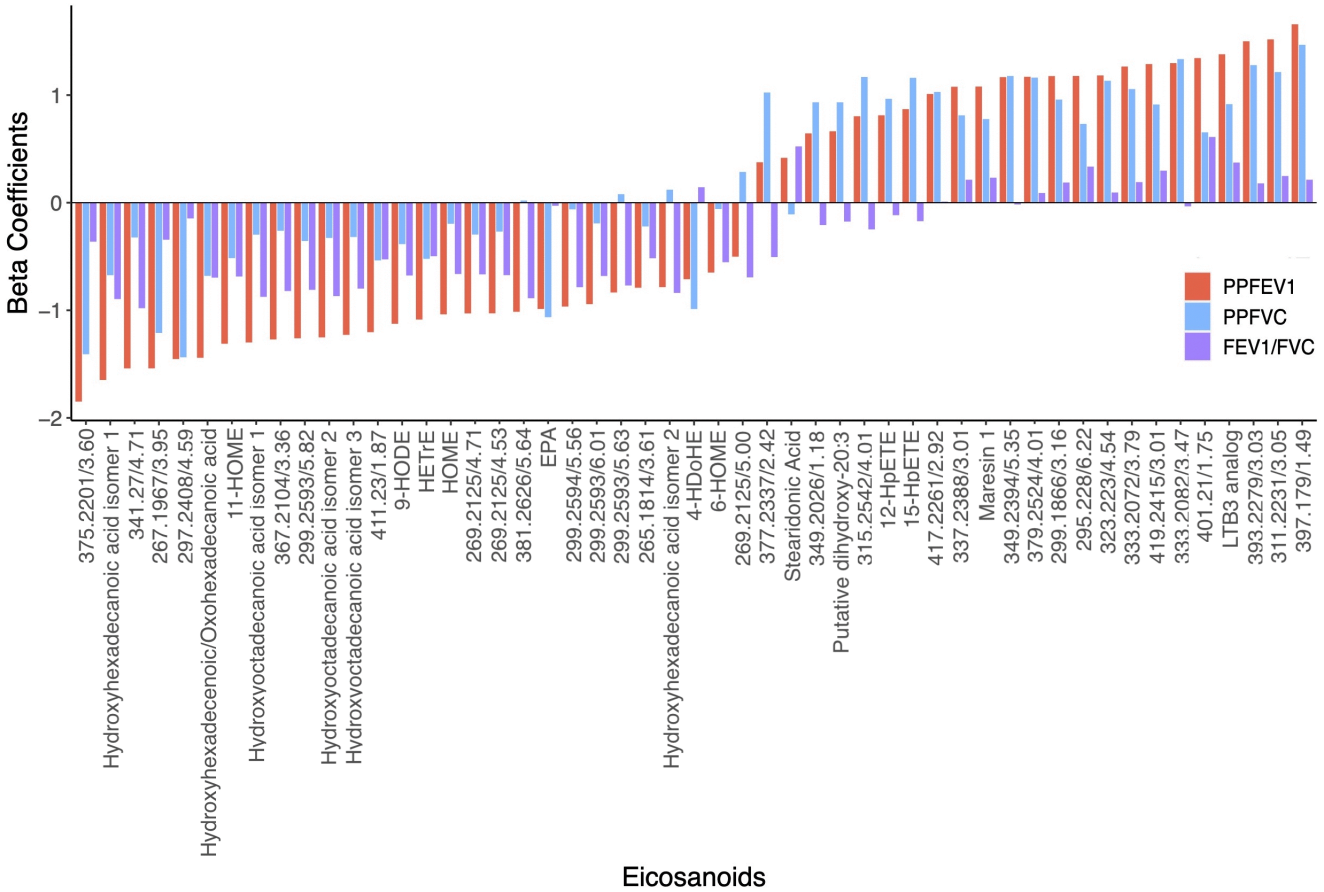
Waterfall plot of the association of eicosanoids with lung function. Associations with PPFEV1 (red), PPFVC (blue), and FEV1/FVC (purple) are shown. 51 metabolites displayed at least one significant association with spirometry traits. Beta estimates represent multivariable-adjusted associations between eicosanoids and spirometry variables. Metabolites are designated with putative ID if known and mass-to-charge ratio/retention time (min) if not.

**Fig 2:**
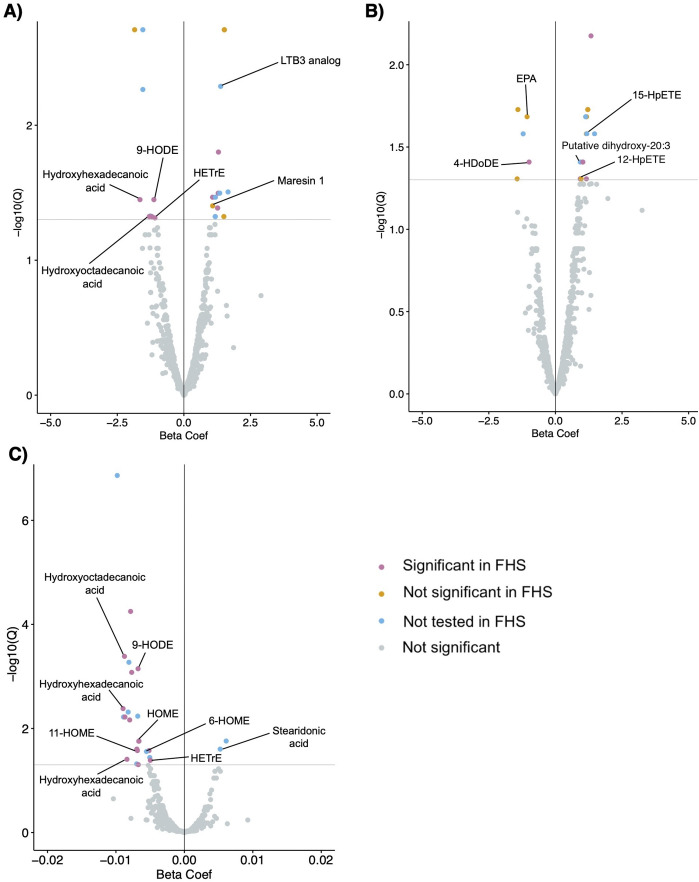
Volcano plots of the associations of eicosanoids with spirometry metrics. Associations with **A)** PPFEV1, **B)** PPFVC, and **C)** FEV1/FVC are shown. Beta coefficients indicate strength of associations. Grey line indicates q < 0.05 with n = 22 significant for PPFEV1, n = 18 for PPFVC, and n = 25 for FEV1/FVC. Coloring indicates those available for validation that did (pink) and did not (orange) validate in FHS.

Of the 784 eicosanoids or related metabolites, 51 were associated with at least one spirometry measure in MESA (FDR q < 0.05, S1 Table, **Fig 1),** with 19 having known molecular identities (**Table 2**). Top metabolites associated with higher PPFEV_1_ included a leukotriene B3 (LTB3) analog and docosahexaenoic acid (DHA) derivative maresin 1 (Fig 2a). By contrast, the linoleic acid derivative 9-hydroxyoctadecadienoic acid (9-HODE), dihomo-gamma linolenic acid (DGLA) derivative hydroxy-eicosatrienoic acid (HETrE), and long-chain fatty acids (LCFAs) including derivatives of palmitic and stearic acid (hydroxyhexadecanoic acid, hydroxyoctadecanoic acids) were associated with lower PPFEV_1_ (Table 2).

**Table 2:** Associations between eicosanoids and spirometry measurements for metabolites with known identities.

			PPFEV1			PPFVC			FEV1/FVC		
Putative Metabolite ID	m/z	Retention time, min	β	SE	p	β	SE	p	β	SE	p
Hydroxyoctadecanoic acid isomer 1	381.2623	5.54	−1.30*	**0.40**	**1.3E-03**	−0.30	0.36	0.41	−0.0088*	**0.0018**	**1.6E-06**
9-HODE	295.228	4.74	−1.13*	**0.33**	**5.5E-04**	−0.38	0.29	0.18	−0.0068*	**0.0015**	**4.5E-06**
Hydroxyhexadecanoic acid isomer 1	353.2313	4.62	−1.65*	**0.48**	**5.9E-04**	−0.67	0.43	0.11	−0.0090*	**0.0022**	**3.7E-05**
Hydroxyoctadecanoic acid isomer 2	381.2622	6.01	−1.25	0.49	1.0E-02	−0.33	0.43	0.45	−0.0087*	**0.0022**	**7.9E-05**
Hydroxyoctadecanoic acid isomer 3	381.2621	5.82	−1.23	0.45	6.9E-03	−0.32	0.40	0.43	−0.0080*	**0.0021**	**1.0E-04**
HOME	379.2515	5.53	−1.04	0.40	0.01	−0.20	0.36	0.59	−0.0066*	**0.0018**	**2.9E-04**
Stearidonic acid	335.2165	6.01	0.42	0.33	0.21	−0.11	0.30	0.71	**0.0052**	**0.0015**	**5.2E-04**
11-HOME	379.2469	4.96	−1.31	0.44	3.1E-03	−0.52	0.39	0.19	−0.0069*	**0.0020**	**6.4E-04**
6-HOME	297.2435	5.36	−0.65	0.36	0.07	−0.06	0.32	0.86	**−0.0055**	**0.0016**	**7.1E-04**
Hydroxyhexadecanoic acid isomer 2	353.2314	4.92	−0.79	0.57	0.17	0.12	0.50	0.81	−0.0084*	**0.0026**	**1.1E-03**
HETrE	321.2438	5.66	−1.09*	**0.34**	**1.4E-03**	−0.52	0.30	0.08	−0.0050*	**0.0015**	**1.2E-03**
Hydroxyhexadecenoic/Oxohexadecanoic acid	351.2151	4.71	−1.44	0.48	2.9E-03	−0.68	0.43	0.11	**−0.0070**	**0.0022**	**1.5E-03**
LTB3 analog	337.2387	4.57	**1.38**	**0.33**	**2.6E-05**	0.92	0.29	1.7E-03	0.0037	0.0015	0.01
Maresin 1	359.2225	3.36	**1.08**	**0.32**	**7.1E-04**	0.78	0.28	6.1E-03	0.0023	0.0014	0.11
Putative dihydroxy-20:3	337.2401	4.84	0.66	0.31	0.03	**0.93**	**0.27**	**6.5E-04**	−0.0018	0.0014	0.21
15-HpETE	335.2233	4.56	0.87	0.36	0.02	**1.16**	**0.32**	**3.2E-04**	−0.0017	0.0016	0.30
4-HDoHE	343.2298	5.43	−0.71	0.33	0.03	−0.99*	**0.29**	**6.7E-04**	0.0014	0.0015	0.33
12-HpETE	335.2195	4.97	0.81	0.33	0.01	**0.97**	**0.30**	**1.1E-03**	−0.0012	0.0015	0.44
EPA	361.2385	6.32	−0.99	0.32	1.8E-03	**−1.06**	**0.28**	**1.5E-04**	−0.0003	0.0014	0.84

β: coefficient of association; Bold: q < 0.05; *FHS-validated.

4-HDoHE: 4-hydroxy-5E,7Z,10Z,13Z,16Z,19Z-docosahexaenoic acid; 6-HOME: 6-hydroxyoctadecenoic acid; 9-HODE: 9-hydroxy-10E,12Z-octadecadienoic acid; 11-HOME: 11-hydroxy-9-octadecenoic acid; 12-HpETE: 12-hydroperoxy-5Z,8Z,10E,14Z-eicosatetraenoic acid; 15-HpETE: 15-hydroperoxy-5Z,8Z,11Z,13E-eicosatetraenoic acid; EPA: 5Z,8Z,11Z,14Z,17Z-eicosapentaenoic acid; HETrE: hydroxyeicosatrienoic acid; HOME: hydroxyoctadecenoic acid; LTB3: leukotriene B3; m/z: mass/charge.

4-HDoHE: 4-hydroxy-5E,7Z,10Z,13Z,16Z,19Z-docosahexaenoic acid; 6-HOME: 6-hydroxyoctadecenoic acid; 9-HODE: 9-hydroxy-10E,12Z-octadecadienoic acid; 11-HOME: 11-hydroxy-9-octadecenoic acid; 12-HpETE: 12-hydroperoxy-5Z,8Z,10E,14Z-eicosatetraenoic acid; 15-HpETE: 15-hydroperoxy-5Z,8Z,11Z,13E-eicosatetraenoic acid; EPA: 5Z,8Z,11Z,14Z,17Z-eicosapentaenoic acid; HETrE: hydroxyeicosatrienoic acid; HOME: hydroxyoctadecenoic acid; LTB3: leukotriene B3; m/z: mass/charge.

Eighteen metabolites were associated with PPFVC in MESA (Fig 2b). Specifically, AA derivatives 15-hydroperoxyeicosatetraenoic acid and 12-hydroperoxyeicosatetraenoic (15-HpETE, 12-HpETE) as well as putative dihydroxy-20:3 were associated with higher PPFVC, while eicosapentaenoic acid (EPA) and DHA derivative 4-hydroxydocosahexaenoic acid (4-HDoHE) were associated with lower PPFVC (Table 2).

Twenty-five metabolites were associated with FEV_1_/FVC ratio in MESA (Fig 2c). Stearidonic acid was associated with greater FEV_1_/FVC ratio. Metabolites that were associated with lower FEV_1_/FVC ratio included palmitic acid derivatives (hydroxyhexadecanoic acid, oxohexadecanoic acid), stearic acid derivative hydroxyoctadecanoic acid, DGLA derivative HETrE, and linoleic acid derivatives (9-HODE, 6-hydroxyoctadecadienoic acid (6-HOME), and 11-hydroxyoctadecenoic acid (11-HOME)) (Table 2).

### Validation of Spirometry-Associated Eicosanoids in FHS

**Fig 3:**
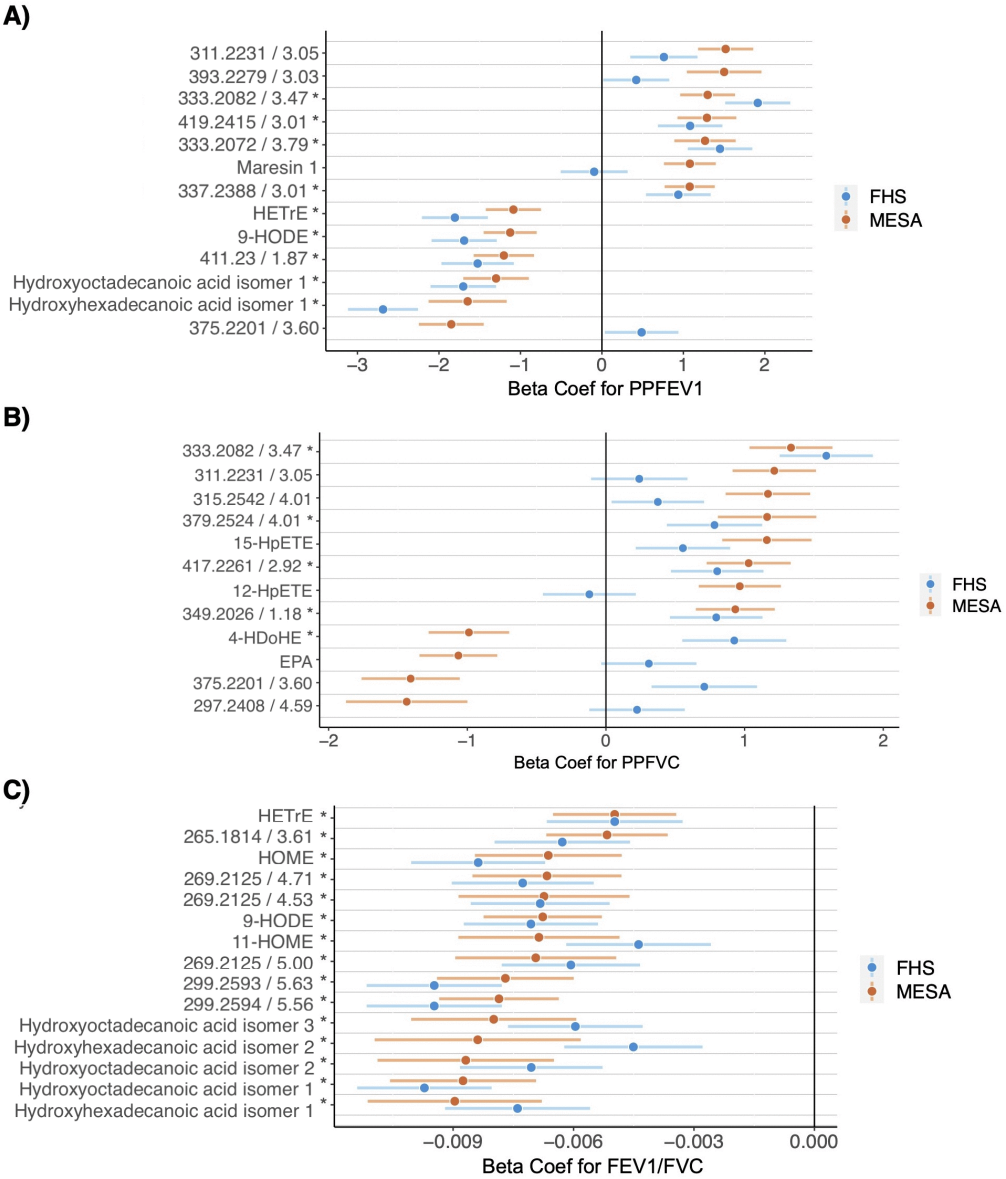
Strength of associations of eicosanoids with spirometry metrics in MESA and FHS cohorts. Eicosanoids significantly associated with **A)** PPFEV1, **B)** PPFVC, and **C)** FEV1/FVC in MESA (orange) and available for validation in FHS (blue) are shown. Beta estimates represent strength of associations. Metabolites are designated with putative ID if known and mass-to-charge ratio/retention time (min) if not. *Validated in FHS participants.

Among 22 metabolites associated with PPFEV_1_ in MESA, 13 were available for validation in FHS, with 9 validating (Fig 2a). Directionality was consistent for all PPFEV_1_ associated metabolites that validated (**Fig 3a**) and included the following metabolites: 9-HODE, hydroxyhexadecanoic acid, hydroxyoctadecanoic acid, and HETrE, all associated with lower PPFEV_1_ across both MESA and FHS.

Among 18 metabolites associated with PPFVC in MESA, 12 were available in FHS and 5 validated (Fig 2b). Validated metabolites were directionally concordant, excepting DHA derivative 4-HDoHE which was associated with lower PPFVC in MESA and higher PPFVC in FHS (Fig 3).

Lastly, among 25 metabolites associated with FEV_1_/FVC in MESA, 15 were available in FHS, and all validated, with all metabolites associated with lower FEV_1_/FVC across both cohorts (**Fig 3c**).

We examined associations with DLCO in FHS. Among 51 metabolites associated with at least one spirometry measure in MESA, 33 were available in FHS, with 18 associated with DLCO. Thirteen metabolites were associated with lower DLCO and directionally consistent across other phenotypes, including linoleic acid derivatives 9-HODE and 11-HOME and palmitic and stearic acid derivatives hydroxyhexadecanoic and hydroxyoctadecanoic acids, which were associated with lower PPFEV_1_ and lower FEV_1_/FVC, as well as AA derivative 12-HpETE which was associated with greater PPFVC. (S1 Table).

### Associations of Significant Eicosanoids with Obstructive and Restrictive Physiology

Of the 51 metabolites that were associated with spirometry measures, 27 were associated with obstructive and 14 with restrictive physiology in MESA (Table 3A-3B).

**Table 3:** Associations between eicosanoids and obstructive and restrictive lung disease.

Putative Metabolite ID	m/z	Retention time, min	OR	95% CI	P
**A. Obstructive Disease**					
*Associated with higher odds*					
Unknown	341.27	4.71	1.35	(1.25, 1.44)	2.0E-09
Unknown	375.2201	3.60	1.25	(1.12, 1.38)	6.3E-04
Hydroxyhexadecanoic acid isomer 1*	353.2313	4.62	1.25	(1.11, 1.40)	2.4E-03
Unknown	367.2104	3.36	1.24	(1.08, 1.40)	8.9E-03
Hydroxyhexadecenoic/Oxohexadecanoic acid	351.2151	4.71	1.22	(1.07, 1.37)	8.9E-03
Unknown	299.2593	5.82	1.21	(1.10, 1.33)	8.7E-04
Hydroxyoctadecanoic acid isomer 1*	381.2623	5.54	1.21	(1.10, 1.33)	9.9E-04
Unknown*	269.2125	4.71	1.21	(1.09, 1.34)	2.2E-03
Unknown*	269.2125	4.53	1.21	(1.07, 1.35)	8.6E-03
Hydroxyoctadecanoic acid isomer 2*	381.2622	6.01	1.21	(1.06, 1.36)	0.01
Unknown*	299.2594	5.56	1.19	(1.10, 1.28)	1.8E-04
Unknown*	299.2593	5.63	1.19	(1.08, 1.30)	1.5E-03
Unknown*	265.1814	3.61	1.19	(1.08, 1.30)	1.6E-03
Unknown	381.2626	5.64	1.19	(1.04, 1.34)	0.02
Hydroxyoctadecanoic acid isomer 3*	381.2621	5.82	1.18	(1.04, 1.32)	0.02
9-HODE*	295.228	4.74	1.17	(1.08, 1.27)	1.1E-03
Unknown	299.2593	6.01	1.17	(1.05, 1.28)	7.4E-03
Unknown*	269.2125	5.00	1.17	(1.04, 1.31)	0.02
Unknown	267.1967	3.95	1.15	(1.04, 1.27)	0.01
6-HOME	297.2435	5.36	1.14	(1.03, 1.26)	0.02
Unknown*	411.23	1.87	1.14	(1.02, 1.25)	0.03
*Associated with lower odds*					
LTB3 analog	337.2387	4.57	0.89	(0.79, 0.99)	0.02
Stearidonic Acid	335.2165	6.01	0.88	(0.78, 0.98)	0.01
Maresin 1	359.2225	3.36	0.88	(0.78, 0.99)	0.02
Unknown	295.228	6.22	0.86	(0.75, 0.98)	0.01
Unknown	397.179	1.49	0.86	(0.72, 1.00)	0.03
Unknown	401.21	1.75	0.84	(0.73, 0.96)	3.5E-03
**B. Restrictive Disease**					
*Associated with higher odds*					
EPA	361.2385	6.32	1.21	(1.09, 1.34)	3.0E-03
Stearidonic Acid	335.2165	6.01	1.20	(1.06, 1.35)	0.01
Unknown	267.1967	3.95	1.19	(1.06, 1.33)	9.4E-03
*Associated with lower odds*					
Unknown*	417.2261	2.92	0.86	(0.73, 0.99)	0.02
Unknown	299.1866	3.16	0.85	(0.72, 0.97)	0.01
Unknown	311.2231	3.05	0.85	(0.71, 0.99)	0.02
Unknown	323.223	4.54	0.85	(0.72, 0.98)	0.02
Unknown	333.2072	3.79	0.84	(0.70, 0.99)	0.02
Putative dihydroxy-20:3	337.2401	4.84	0.83	(0.69, 0.97)	7.4E-03
LTB3 analog	337.2387	4.57	0.83	(0.69, 0.97)	7.9E-03
Unknown	377.2337	2.42	0.82	(0.68, 0.95)	2.9E-03
Unknown	349.2394	5.35	0.82	(0.67, 0.96)	5.6E-03
Unknown*	333.2082	3.47	0.81	(0.68, 0.93)	5.9E-04
Unknown*	393.2279	3.03	0.75	(0.55, 0.94)	3.0E-03

Significance defined as FDR q < 0.1. Putative metabolite IDs shown for metabolites with known confirmed identities.

*Validated in FHS participants.

6-HOME: 6-hydroxyoctadecenoic acid; 9-HODE: 9-hydroxy-10E,12Z-octadecadienoic acid; EPA: 5Z,8Z,11Z,14Z,17Z-eicosapentaenoic acid; LTB3: leukotriene B3; m/z: mass/charge; OR: odds ratio.

**Fig 4:**
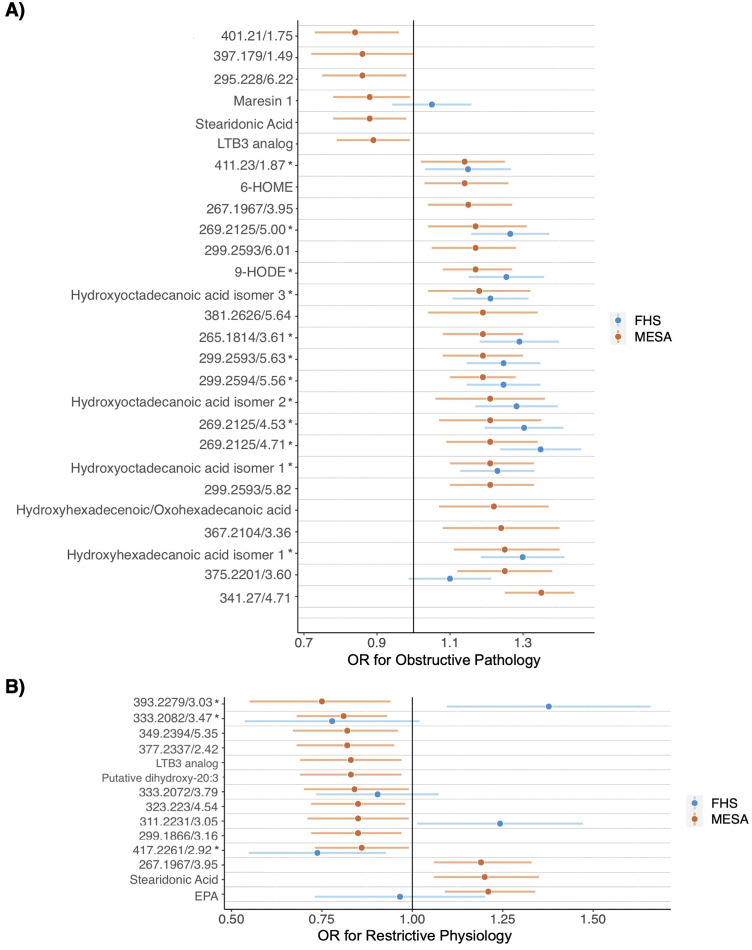
Associations of eicosanoids with obstructive and restrictive lung physiology in MESA and FHS cohorts. Associations with **A**) obstructive or **B**) restrictive physiology in MESA (orange) and FHS (blue) are shown. Odds ratios represent odds of obstructive vs normal, or restrictive vs normal, per 1-SD higher log-transformed metabolite. Metabolites are designated with putative ID if known and mass-to-charge/retention time (min) if not. *Validated in FHS.

**Fig 5:**
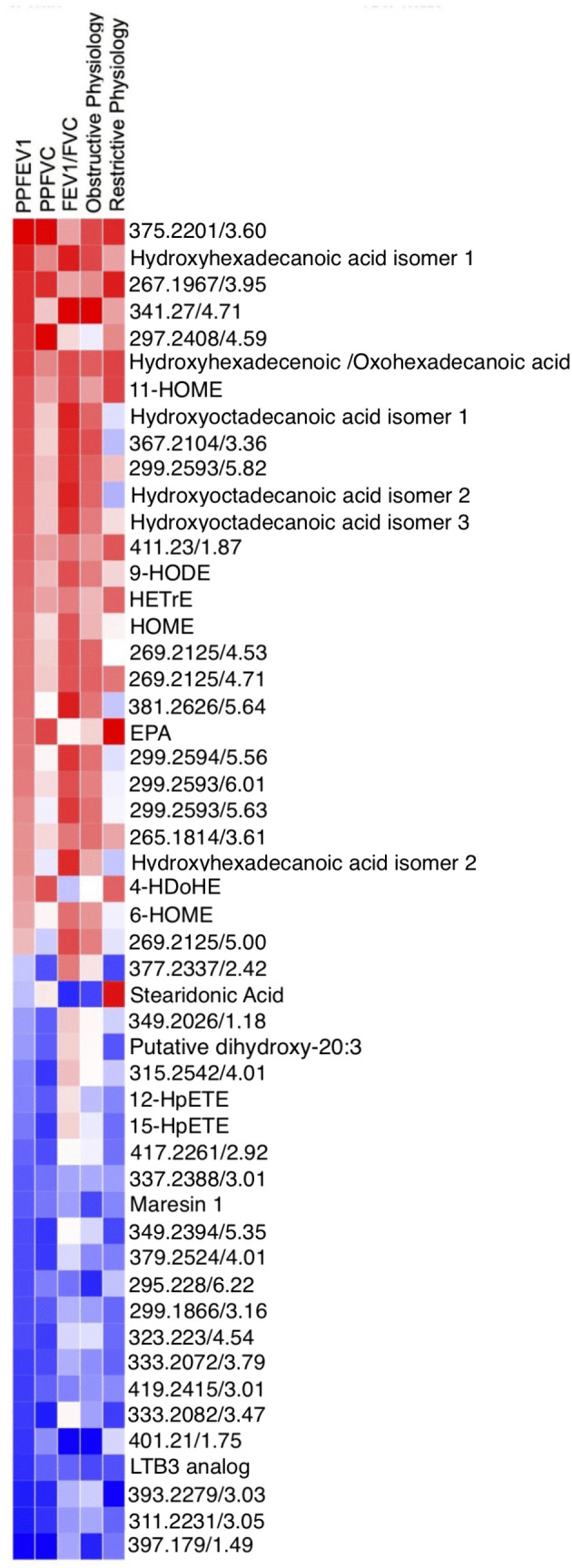
Heatmap illustrating directionality for associations of eicosanoids and related metabolites with lung function traits. Associations with PPFEV1, PPFVC, FEV1/FVC ratio, obstructive physiology, and restrictive physiology are shown. Eicosanoid metabolite are annotated by name or mass to charge ratio and retention time. Color scale represents beta coefficients for continuous traits and odds ratios for obstructive and restrictive physiology, scaled to maximum and minimum values, with red indicating worse lung function and blue indicating better lung function (heatmap created using Morpheus, https://software.broadinstitute.org/morpheus).

Of the 27 metabolites associated with obstructive physiology, 14 were available in FHS, and 12 replicated as significant associations. Metabolites with significant associations in both MESA and FHS included 9-HODE, a hydroxyhexadecanoic acid isomer, and isomers of hydroxyoctadecanoic acid, all of which were associated with greater odds of obstructive physiology. Specifically, a 1-standard deviation (SD) higher hydroxyoctadecanoic acid isomer 1 was associated with 1.21-fold (95% CI 1.10, 1.33) greater odds of obstruction. Similarly, a 1-SD higher 9-HODE was associated with 1.17-fold (95% CI 1.08, 1.27) higher odds of obstruction (Fig 4A). Most metabolites with significant associations in only MESA participants were also associated with greater odds of obstruction, including stearidonic acid and 6-HOME. Only 6 metabolites were associated with lower odds of obstructive physiology, including an LTB3 analog and maresin 1 (Fig 5).

Of the 14 metabolites associated with restrictive physiology in MESA, 6 were available in FHS, and 3 replicated as significant associations. Although molecular identities of these 3 metabolites were unknown, all 3 were associated with lower odds of restrictive physiology. Most of the metabolites associated with restrictive physiology in only MESA participants were also associated with lower odds, including putative dihydroxy-20:3 and LTB3 analog (OR 0.83, 95% CI 0.69, 0.97). By contrast, stearidonic acid and EPA were associated with greater odds of restrictive physiology (OR 1.20, 95% CI 1.06–1.35; OR 1.21, 95% CI 1.09, 1.34, respectively; **Fig 4B**).

### Smoking status and the association of eicosanoids with obstructive physiology

Of the 3384 MESA participants, 354 (10.5%) were current and 1288 (38.1%) former smokers. Twenty-six of 51 metabolites from primary analyses remained significantly associated with at least one spirometry variable after additionally adjusting for smoking status in secondary analysis (S2 Table). Further, effect sizes and directionality of associations were similar after addition of smoking status (S2 Fig).

To examine whether eicosanoid metabolites may mediate the association of smoking with obstructive physiology, we conducted mediation analyses. Out of the 27 metabolites associated with obstructive physiology in MESA, we found that smoking status mediated the association of 5 metabolites with obstructive physiology (S3 Table). This included 9-HODE (0.90% effect mediated), maresin 1 (3.3%), and a hydroxyoctadecanoic acid isomer (2.6%). Most metabolites demonstrated mediation effects of small magnitudes, with the largest effect being approximately 4%.

### Associations of significant eicosanoids with lung imaging features

Among 2233 MESA participants with available CT scans, mean % emphysema was 4.1 ± 3.9, mean % HLA 6.8 ± 4.5, and 586 (22.6%) had ILA. As expected, % emphysema was higher among patients with obstructive physiology (grade 1: 6.1 ± 4.4, grade 2–4: 6.1 ± 5.4) compared to those with normal or restrictive physiology (3.8 ± 3.7; 2.3 ± 2.3). Similarly, % HAA was higher among participants with restrictive physiology (8.9 ± 6.5) compared to those with obstructive physiology (grade 1: 5.7 ± 2.9, grade 2–4: 5.8 ± 2.2) (**Table 1**).

Of the 51 metabolites associated with spirometry measures in MESA, 5 were associated with % emphysema, including LTB3 analog (previously associated with higher PPFEV_1_) which was associated with lower % emphysema (**Table 4A**). Six metabolites were associated with % HAA (Table 4A-4C), including HETrE, putative dihydroxy-20:3, and LTB3 analog, which were associated with greater % HAA, and 11-HOME which was associated with lower % HAA. Three metabolites were associated with ILA yet did not have confirmed known identities.

**Table 4:** Associations between eicosanoids and lung imaging features.

A: Percent Emphysema					
Putative Metabolite ID	m/z	Retention time, min	β	SE	P
Unknown	341.27	4.71	−0.29	0.09	7.5E-04
Unknown	411.23	1.87	0.26	0.09	0.00
Unknown	299.1866	3.16	0.23	0.09	0.01
Unknown	401.21	1.75	−0.22	0.09	0.01
LTB3 analog	337.2387	4.57	−0.18	0.08	0.04
**B: HAA Continuous**					
**Putative Metabolite ID**	**m/z**	**Retention time, min**	**β**	**SE**	**P**
Unknown	401.21	1.75	0.04	0.01	1.3E-03
HETrE	321.2438	5.66	0.03	0.01	3.4E-02
Putative dihydroxy-20:3	337.2401	4.84	0.03	0.01	0.02
LTB3 analog	337.2387	4.57	0.03	0.01	0.02
Unknown	341.27	4.71	0.03	0.01	0.05
11-HOME	379.2469	4.96	−0.04	0.02	0.04
**C: HAA Dichotomized**					
**Putative Metabolite ID**	**m/z**	**Retention time, min**	**OR**	**95% CI**	**P**
Unknown	299.1866	3.16	0.81	(0.68, 0.94)	0.00
Unknown	299.2593	6.01	0.83	(0.69, 0.97)	0.01
11-HOME	379.2469	4.96	0.80	(0.63, 0.97)	0.01
Hydroxyoctadecanoic acid isomer 2	381.2622	6.01	0.78	(0.59, 0.96)	0.01
Unknown	401.21	1.75	1.17	(1.04, 1.31)	0.02
Putative dihydroxy-20:3	337.2401	4.84	1.13	(1.03, 1.24)	0.02
Unknown	311.2231	3.05	0.87	(0.75, 0.99)	0.03
6-HOME	297.2435	5.36	0.87	(0.74, 1.01)	0.05
Unknown	299.2593	5.82	0.86	(0.72, 1.01)	0.05
Hydroxyhexadecanoic acid isomer 1	353.2313	4.62	0.84	(0.66, 1.01)	0.05
**D: ILA**					
**Putative Metabolite ID**	**m/z**	**Retention time, min**	**OR**	**95% CI**	**P**
Unknown	299.1866	3.16	0.85	(0.72, 0.97)	0.01
Unknown	299.2594	5.56	1.16	(1.05, 1.28)	0.01
Unknown	299.2593	5.63	1.14	(1.01, 1.27)	0.05

Significance defined as p < 0.05. Putative metabolite IDs shown for metabolites with known confirmed identities.

β: coefficient of association; 6-HOME: 6-hydroxyoctadecenoic acid; 11-HOME: 11-hydroxy-9-octadecenoic acid; HETrE: hydroxyeicosatrienoic acid; LTB3: leukotriene B3; m/z: mass-to-charge ratio; OR: odds ratio.

## Discussion

We performed molecular profiling of eicosanoids and eicosanoid-related metabolites to evaluate associations with lung function across two large community-based samples. Our findings are as follows: First, we identified 51 eicosanoids and related metabolites associated with spirometry measures, including 27 associated with obstructive and 14 with restrictive physiology. This included linoleic acid derivatives (9-HODE) and LCFAs including palmitic and stearic acid derivatives (hydroxyhexadecanoic acid, hydroxyoctadecanoic acid), which were associated with lower PPFEV_1_, FEV_1_/FVC, and odds of obstructive physiology across both samples. Fewer metabolites were associated with restrictive physiology, and included an LTB3 analog, associated with higher PPFEV_1_, lower odds of restriction, and lower % emphysema. These findings highlight the role of specific bioactive lipids associated with early changes in lung function.

### Associations with obstructive lung physiology

The majority of metabolites identified were associated with obstructive physiology, with expected overlapping associations with PPFEV_1_ and FEV_1_/FVC. Interestingly, most metabolites were associated with higher odds of obstruction, including 9-HODE, a pro-inflammatory derivative of linoleic acid previously associated in lung injury and airway inflammation. In studies of porcine models with surfactant depletion, lung injury induced through hyperinflation was shown to result in higher levels of oxylipins including 9-HODE [39]. Similarly, in a study of hospitalized COVID-19 patients and negative controls, exhaled breath condensate samples from COVID-19 patients showed significantly higher levels of eicosanoid compounds including 9-HODE, proposing a possible mechanism for the lung injury and respiratory tract damage seen in severe COVID-19 cases [40]. Experimental data have evaluated potential mechanisms, including in vitro models in which both human and bovine polymorphonuclear leukocytes showed a chemotactic response to 9-HODE, suggesting that 9-HODE stimulates recruitment of pro-inflammatory mediators into airways [41]. We expand upon these findings and show robust association of 9-HODE levels with obstructive lung physiology, an effect that appears in part mediated by smoking status, consistent with the hypothesis that inhalation insults contribute to downstream activation of inflammatory pathways that may adversely affect lung function.

Similarly, we found two LCFAs associated with obstructive lung physiology. Specifically, hydroxyoctadecenoic acid, an unsaturated fatty acid, and isomers of its derivative hydroxyoctadecanoic acid were associated with lower FEV_1_/FVC and higher odds of obstructive physiology in both MESA and FHS. As a naturally-occurring ligand for peroxisome proliferator-activated receptor γ (PPARγ), hydroxyoctadecanoic acid has been shown to inhibit surfactant protein B gene expression in the lung, affecting surfactant homeostasis [42]. While patients with chronic asthma are thought to have normal baseline surfactant, in exacerbations the disruption of surfactant homeostasis is thought to contribute to symptoms. Cigarette smoking and COPD also lead to surfactant dysregulation, contributing to lung function decline [43]. Lastly, we found that isomers of a separate LCFA, hydroxyhexadecanoic acid, were also associated in both MESA and FHS with a low FEV_1_/FVC and an obstructive physiology, although their associations with lung function have not been studied and may represent an avenue for future investigation.

### Associations with restrictive lung physiology

While we found 14 metabolites associated with restrictive physiology in MESA, only three that were associated with lower odds validated in FHS. All three were novel putative eicosanoids with unknown exact molecular identity. Within MESA, we found that EPA, an omega-3 polyunsaturated fatty acid, was associated with higher odds of restrictive ventilatory deficit in MESA and FHS. EPA, converted from alpha linolenic acid or obtained via dietary intake, is anti-inflammatory via inhibition of AA and function as precursors to pro-resolving mediators [44]. Omega-3 fatty acids have been shown to mitigate injury from cigarette smoke induced lung inflammation, bleomycin induced pulmonary fibrosis in mice, and mouse models of acute respiratory distress syndrome [45–47]. Additionally, in a prior study of participants in MESA with interstitial lung disease (ILD), circulating higher levels omega-3 fatty acids were associated with a lower risk of adverse outcomes as well as in healthy MESA participants a slower rate of lung function decline [48, 49]. Although it is not clear why we found an association between elevated levels of these omega-3 fatty acids and increased odds of restrictive physiology, it is possible that elevated levels of these anti-inflammatory mediators may have disease mitigating and possibly protective effects in this ostensibly healthy population with presumed early stage disease. However, further research is needed regarding the specific mechanism underlying ILD and the role of elevated levels of omega-3 fatty acids.

### Associations with lung structure

With respect to overlapping findings with lung structure, we found consistent associations of leukotrienes with CT-based lung imaging measures. Specifically, putative dihydroxy-20:3 and LTB3 analog were associated with greater odds of having HAA, a measurement of subclinical ILD. A pro-inflammatory metabolite derived from AA, LTB3 has not yet been implicated in lung disease, however LTB4, with equipotent pro-inflammatory effects, has been implicated in neutrophilic pulmonary inflammation and pathogenesis of murine emphysema [50]. Interestingly, HAA have been associated with other biomarkers of inflammation including matrix metalloproteinase-7 and IL-6 [32], and our results support the role of LTB3 as a potential contributor. However, while trace formation of LTB3 cannot be excluded, endogenous production of LTB3 in a well-nourished human populations is considered negligible due to the the low abundance of its precursor mead acid and the inefficiency of LTA3 conversion to LTB3 in an in vivo setting without essential fatty acid deficiency [51]. Accordingly, repeated detection of an LTB3-annotated feature in this cohort may reflect an alternative dihydroxyeicosanoid such as dihydro-LTB4, which is abundantly produced in leukocytes, or a structurally related isomer rather than authentic LTB3.

### Limitations

Our study has limitations worth noting. Given this was an observational cross-sectional study we are unable to infer causal relationships between eicosanoid pathways and lung diseases. Longitudinal sampling of eicosanoids and lung function, especially in response to anti-inflammatory medications, would make identification of such causal relationships more feasible. Second, lipidomic profiling was performed on samples collected one exam cycle before spirometry was performed in MESA, thus temporal and selection biases are possible limitations. Additionally, plasma samples were collected approximately twenty years ago and stored at −80°C until the time of lipidomic profiling. All samples underwent at most one freeze-thaw cycle until the time of assay, at which time the sample was thawed and aliquoted for LC-MS analysis. Therefore, it is important to acknowledge the potential impacts of non-enzymatic autoxidation of the lipids [52]. and a prior freeze-thaw cycle on the molecular profiling results. Further, some eicosanoids associated with lung function were novel molecules, whose molecular identities remain to be determined in future chemistry-based studies beyond the scope of this analysis. Compound assignment in this study was also based on nominal mase alone and future follow-up work to apply full MS/MS-based structural confirmation is needed. Diffusion capacity was not available in MESA, limiting inferences based on DLCO in this sample, though complementary lung imaging findings were ascertained. We acknowledge that FHS participants were predominantly white, limiting generalizability of the validation sample, though the MESA sample was more diverse.

## Conclusions

Across two large community-based samples of ostensibly healthy adults, we found 51 eicosanoid and eicosanoid-related metabolites associated with lung structure and function. Specifically pro-inflammatory linoleic acid derivatives and LCFAs known to affect cellular signaling and surfactant production were associated with obstructive physiology. Further, putative pro-inflammatory dihydroxy-20:3 and an analog to LTB3 were associated with emphysema and HAA on lung imaging. These findings highlight bioactive lipid pathways that may play a role in the development of specific lung diseases, and may provide insights into disease pathophysiology and future development of therapeutic interventions.

## Supporting information

S1 Fig Timing of data collection and sample selection criteria for MESA and FHS cohorts. Final samples used for analysis shown in orange (MESA) and blue (FHS).
(DOCX)

S1 Table Full table of associations of all 51 significant metabolites with spirometry measures in MESA and FHS. (XLSX)

S2 Table Associations of 51 significant metabolites with spirometry measures after additionally adjusting for smoking status. (XLSX)

S2 Fig Relationships between beta coefficients of primary analysis multivariable linear regression model with beta coefficients of models additionally adjusted for smoking status for PPFEV_1_ (A), PPFVC (B), and FEV_1_/FVC (C) models. In general, effect sizes are slightly reduced after adjusting for smoking status.
(DOCX)

S3 Table Mediation effect of smoking status on association between metabolites and obstructive physiology. (XLSX)

S1 Checklist Human participants research checklist. (DOCX)
