# Supplementary material for: The association of eicosanoids with lung structure and function: Findings from the Multi-Ethnic Study of Atherosclerosis lung study and Framingham Heart Study
Source: PLoS One. 2026 Jun 30;21(6):e0351692. doi: 10.1371/journal.pone.0351692 (PMC13318034; doi:10.1371/journal.pone.0351692)
Supplement: S1 Fig — Final samples used for analysis shown in orange (MESA) and blue (FHS). (DOCX) [file pone.0351692.s001.docx]

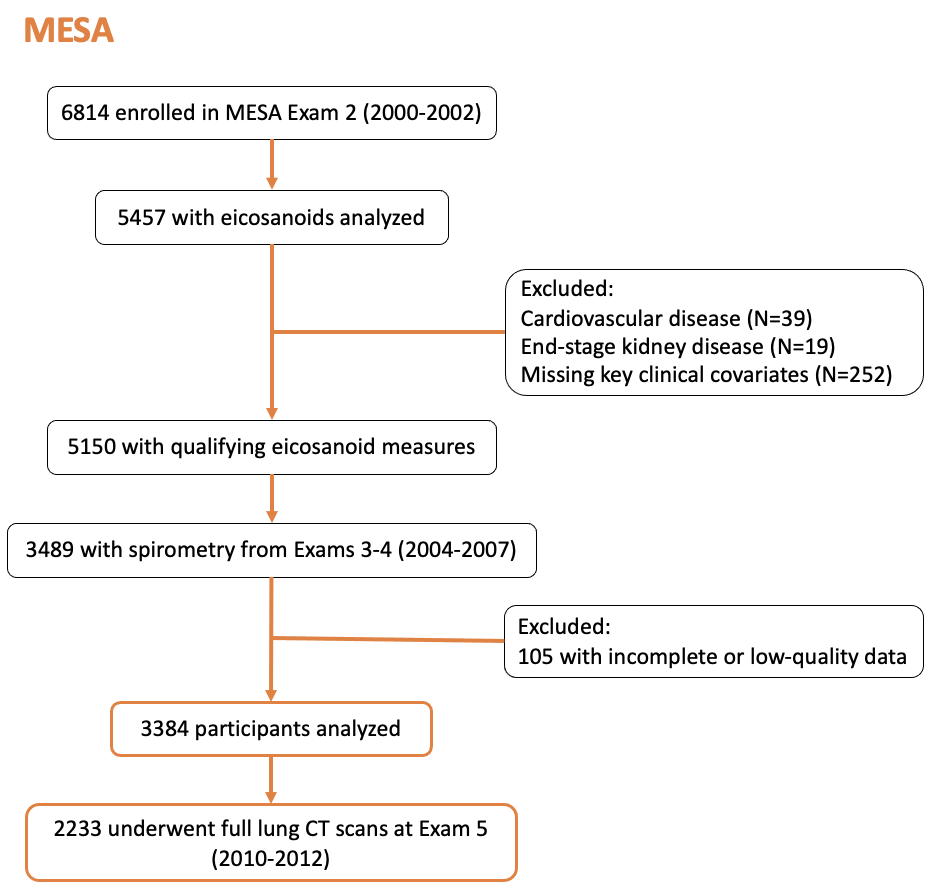


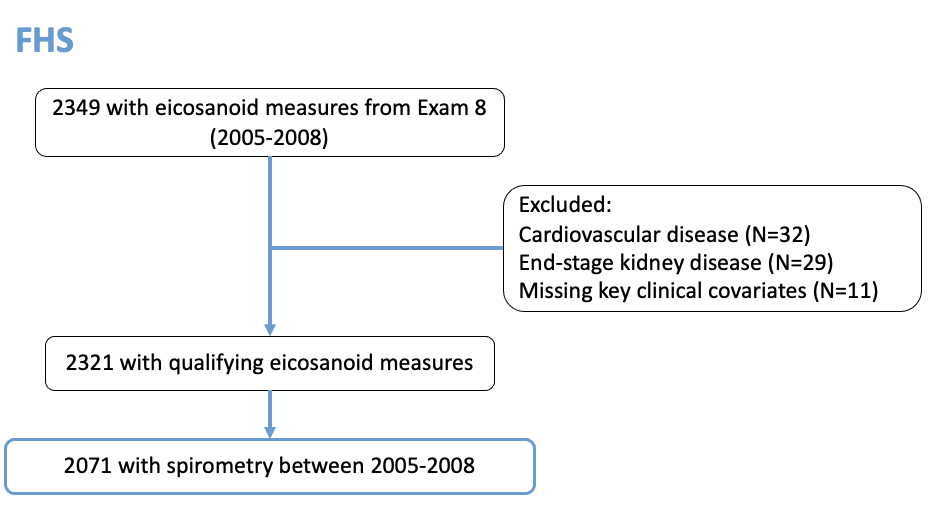


**S1 Figure:** Timing of data collection and sample selection criteria for MESA and FHS cohorts. Final samples used for analysis shown in orange (MESA) and blue (FHS).
