# Supplementary material for: The association of eicosanoids with lung structure and function: Findings from the Multi-Ethnic Study of Atherosclerosis lung study and Framingham Heart Study
Source: PLoS One. 2026 Jun 30;21(6):e0351692. doi: 10.1371/journal.pone.0351692 (PMC13318034; doi:10.1371/journal.pone.0351692)
Supplement: S2 Fig — In general, effect sizes are slightly reduced after adjusting for smoking status. (DOCX) [file pone.0351692.s004.docx]

| **A)**  **** | **B)**  **** |
| --- | --- |
| **C)**  **** | |

**S2 Figure:** Relationships between beta coefficients of primary analysis multivariable linear regression model with beta coefficients of models additionally adjusted for smoking status for PPFEV_1_ (A), PPFVC (B), and FEV_1_/FVC (C) models. In general, effect sizes are slightly reduced after adjusting for smoking status.
