# Supplementary material for: The association of eicosanoids with lung structure and function: Findings from the Multi-Ethnic Study of Atherosclerosis lung study and Framingham Heart Study
Source: PLoS One. 2026 Jun 30;21(6):e0351692. doi: 10.1371/journal.pone.0351692 (PMC13318034; doi:10.1371/journal.pone.0351692)
Supplement: S1 Checklist — (DOCX) [file pone.0351692.s006.docx]

**Human Participants Research Checklist**

***Complete the following if your study involved human participants or human participants’ data. These questions should be addressed for prospective and retrospective studies.***

1. Did you obtain ethics approval for this study?
   - If yes, please upload (file type “Other”) all the approval documents you received from your ethics committee to cover the entire range of the study period (i.e. the original approval document and any extension documents). Where ethics approval was obtained from more than one study location, please provide approval document(s) from all of the sites. If the original document is in another language, please also provide an English translation.

__x_ Uploaded ___ N/A

- - If you did not obtain ethical approval, please explain why this was not required below.

This retrospective study represents secondary use of an existing deidentified and coded dataset. We have obtained and uploaded IRB approval documents from BIDMC (starting 2022 through present) to demonstrate ethics approval required for our study as requested.

By way of background, the existing dataset came from parent studies that are long-standing large NIH-funded studies (Multi-Ethnic Study of Atherosclerosis and Framingham Heart Study). These are now publicly available via dbGAP as outlined in the submission. We did not receive any identifiable information, nor was there further participant recruitment or enrollment as part of our study, and all of the work in the present manuscript was covered by our BIDMC ethics approval as uploaded above.

Please note that we have included informed consent form and current IRB approval for the parent studies (MESA and FHS), along with documentation from the study leadership that the IRB study activities have been approved without lapse since inception of the study. We have forwarded e-mail communication with study leadership to the editorial office as well.

1. If you prospectively recruited human participants for the study – for example, you conducted a clinical trial, distributed questionnaires, or obtained tissues, data or samples for the purposes of this study, please report in the Methods:
   1. the day, month and year of the **start and end** of the recruitment period for this study.
   2. whether participants provided informed consent, and if so, what type was obtained (for instance, written or verbal, and if verbal, how it was documented and witnessed). If your study included minors, state whether you obtained consent from parents or guardians. If the need for consent was waived by the ethics committee, please include this information.

Please state the line number(s) in the Methods where this is reported ______

___ Completed __x_ N/A

**No prospective recruitment was performed as part of this study.**

1. If you are reporting a retrospective study of, for example, medical records, archived samples, survey data, please report in the Methods section:
2. the day, month and year when the data were accessed for research purposes
3. whether authors had access to information that could identify individual participants during or after data collection

Please state the line number(s) in the Methods where this is reported __page 6, line 3____

__x_ Completed ___ N/A

**This was a retrospective study and secondary use of an existing dataset – we have indicated the following in the methods section:**

**“Our study involved secondary use study of existing deidentified and coded data accessed on 08/01/2022 and was approved by the Beth Israel Deaconess Medical Center institutional review board. Investigators did not have access to information that could identify individual participants during or after data collection.”**
